# Molecular recognition of isomeric protonated amino acid esters monitored by ESI-mass spectrometry

**DOI:** 10.3762/bjoc.10.78

**Published:** 2014-04-09

**Authors:** Andrea Liesenfeld, Arne Lützen

**Affiliations:** 1 University of Bonn, Kekulé-Institute of Organic Chemistry and Biochemistry, Gerhard-Domagk-Str.1, D-53121 Bonn, Germany

**Keywords:** amino acids, isomer labelled guest method (ILGM), mass spectrometry, molecular recognition, 9,9’-spirobifluorenes, template

## Abstract

Two new 9,9’-spirobifluorene-derived crown ethers were prepared and used to recognise constitutionally isomeric amino acid derivatives. The performance of the receptors was evaluated by ESI-mass spectrometry using the isomer labelled guest method (ILGM). This method revealed the preferred binding of L-norleucine and L-leucine compared to L-isoleucine for both receptors. Furthermore, non-covalent isotope effects demonstrate the relevance of dispersive interactions for the overall binding event. These effects also provide hints for the relative spatial orientation of the guest molecules within the host–guest complex, and thereby prove the importance of the spirobifluorene moiety for the observed binding of the protonated amino acid esters.

## Introduction

**Figure 1:**
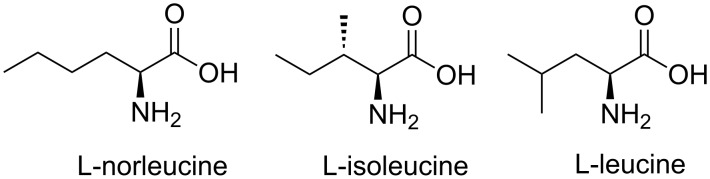
L-Norleucine, L-isoleucine, and L-leucine.

The separation of constitutionally isomeric amino acids is of practical interest. This is particularly true for leucine (Leu), isoleucine (Ile), and norleucine (Nle), especially since the first two are both proteinogenic amino acids (Figure 1). However, it is a great challenge to separate molecules that have the same molecular mass and do not differ significantly in structure. Hence, the chemical and physical properties are very similar, and the isomers leucine, isoleucine, and norleucine are difficult to separate employing commonly used analytical methods like crystallization, enzymatic separation methods, or modified thin-layer chromatography [1–5].

Therefore, we thought to look for a supramolecular approach to achieve amino acid recognition by an artificial host [6–11] with regard to isomer separation upon isomer-selective molecular recognition by a concave template acting as a host, thereby avoiding the necessity to establish or break additional covalent chemical bonds. Hence, we have prepared two new templates based on a 9,9’-spirobifluorene core and tested them with regard to their ability to recognize the three isomeric amino acids in form of their protonated methyl esters. We decided to use ESI-mass spectrometry for these tests since this technique is fast to perform, consumes only trace amounts of material, and can be used to explore competitive experiments that are difficult to perform using UV–vis or NMR spectroscopy.

## Results and Discussion

### Design and synthesis of the concave templates

**Figure 2:**
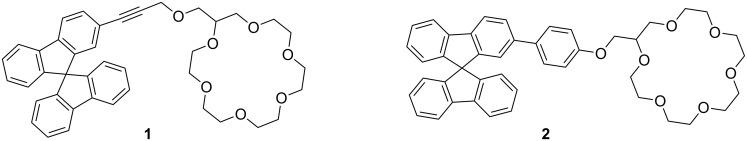
Concave templates **1** and **2**.

Ammonium ions exhibit strong binding affinity towards crown ether moieties. Hence, we decided to use this motif to achieve binding of the leucine isomers as their protonated ester derivatives. This provides the major part of the overall binding energy. However, to distinguish the three isomers the receptors need to provide further elements that either provide additional binding sites for the non-polar parts of the substrates, e.g., via attractive dispersive interactions, or provide steric hindrance that prevents substrates of a certain shape to be accommodated in the concave binding site of the templates. Since the 9,9’-spirobifluorene moiety provides such a rigid concave, non-polar scaffold that has been demonstrated to be a valuable part of some receptors [12–13], we decided to employ this motif. Therefore, we designed the two compounds **1** and **2** shown in Figure 2 that differ only in the bridging element between the crown ether group and the spirobifluorene.

**Scheme 1:**
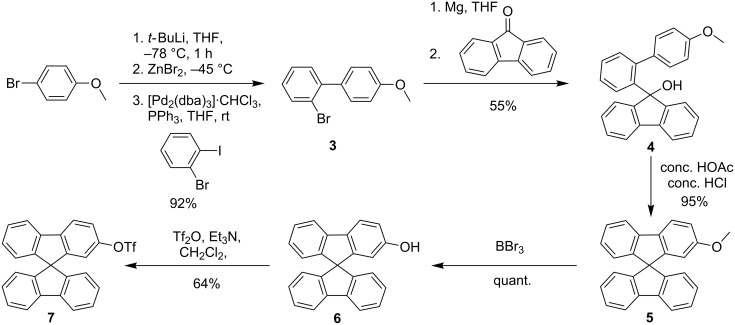
Syntheses of the 2-(9,9’-spirobifluorene-2-yl)trifluoromethansulfonate (**7**).

The synthesis of **1** and **2** started from 1-bromo-4-methoxybenzene which was transferred into 2-bromo-4’-methoxybiphenyl (**3**) in 92% yield via lithiation with *t*-BuLi, transmetallation with zinc(II) bromide, and subsequent Negishi cross-coupling reaction with 1-bromo-2-iodobenzene [14–15]. **3** was then transformed into the corresponding Grignard reagent which was reacted with 9-fluorenone to afford tertiary alcohol **4** in 55% yield. Adopting a protocol of Tour et al. [16] led to 2-methoxy-9,9'-spirobifluorene (**5**) in 95% yield via acidic condensation of **4**. Next, the methoxy group was cleaved quantitatively by reaction with boron tribromide, followed by hydrolysis to afford phenol **6** which was finally transferred into the corresponding triflate **7** in 64% yield (Scheme 1).

**Scheme 2:**
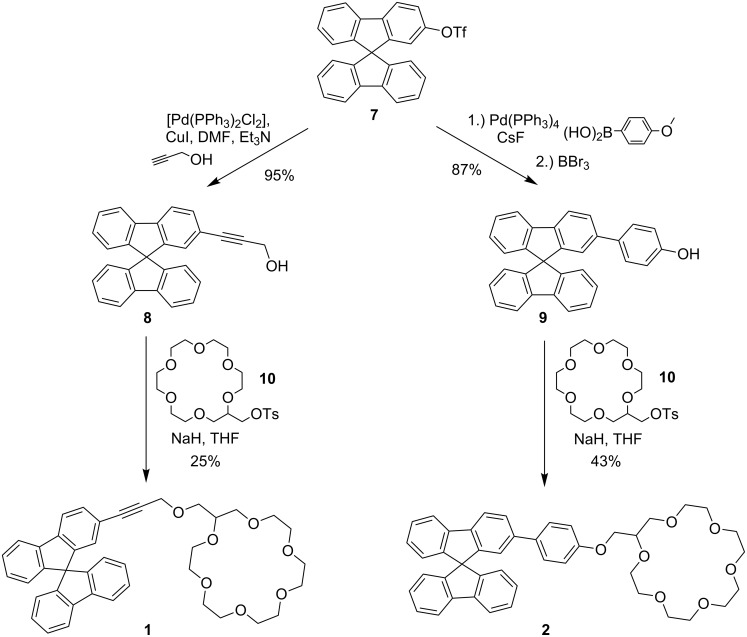
Synthesis of the two receptors **1** and **2**.

Triflate **7** was then subjected to a Sonogashira cross-coupling reaction and a Suzuki cross-coupling reaction followed by treatment with boron tribromide to obtain the ethynylated and arylated alcohols **8** in 95% yield and **9** in 87% yield over both steps, respectively. Finally, deprotonation by sodium hydride and reaction with tosylated 18-crown-6 derivative **10** [17–18] derived from commercially available (1,4,7,10,13,16-hexaoxacyclooctadecan-2-yl)methanol afforded the desired target compounds **1** and **2** in moderate yields (Scheme 2).

### Molecular recognition studies

With our crown ether derivatives **1** and **2** in hands we studied their recognition behaviour towards the L-leucine isomers. Usually, spectroscopic techniques like NMR or UV–vis spectroscopy are used for this purpose. However, mass spectrometry has become a major analytical tool in supramolecular chemistry in recent years [19–21] and seemed to be perfectly suited in this case, since we were planning to recognise the amino acid derivatives in form of their protonated alkyl esters anyway. Thus, the host–guest complexes would be charged and supposedly easy to detect by mass spectrometry if they can be separated from the counter-ions.

**Figure 3:**
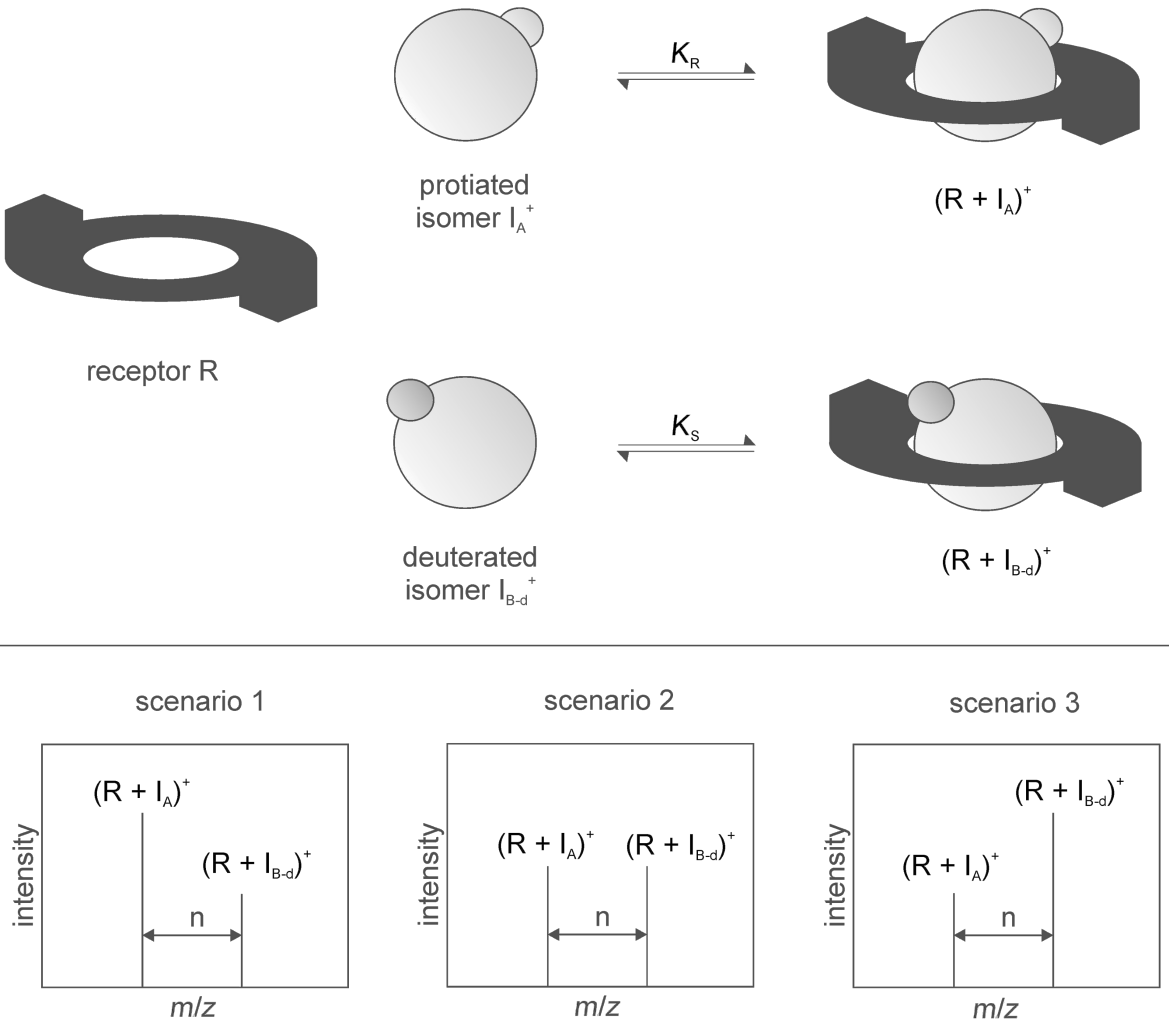
Schematic presentation of the isomer labelled guest method (ILGM).

Nevertheless, it still sounds kind of paradox to use mass spectrometry to study isomeric complexes due to their identical mass/charge ratio. This problem can be circumvented by the use of isotopically labelled substrates in the sense of an isomer labelled guest method (ILGM) (Figure 3) which is closely related to the enantiomer labelled guest method (ELGM) introduced by Sawada [22].

Here, a competitive recognition experiment using a non-labelled substrate and a mass-labelled *quasi*-isomer is performed to reveal the relative affinity of a receptor towards the different isomers. In this way, the mass spectrometric analysis easily allows direct identification of the individual host–guest complexes. This is usually more complicated with other techniques such as, e.g., NMR spectroscopy because it is more difficult to assign the signals to the individual host–guest complexes and the analysis might be additionally hindered or even be impossible due to severe signal overlapping.

**Figure 4:**
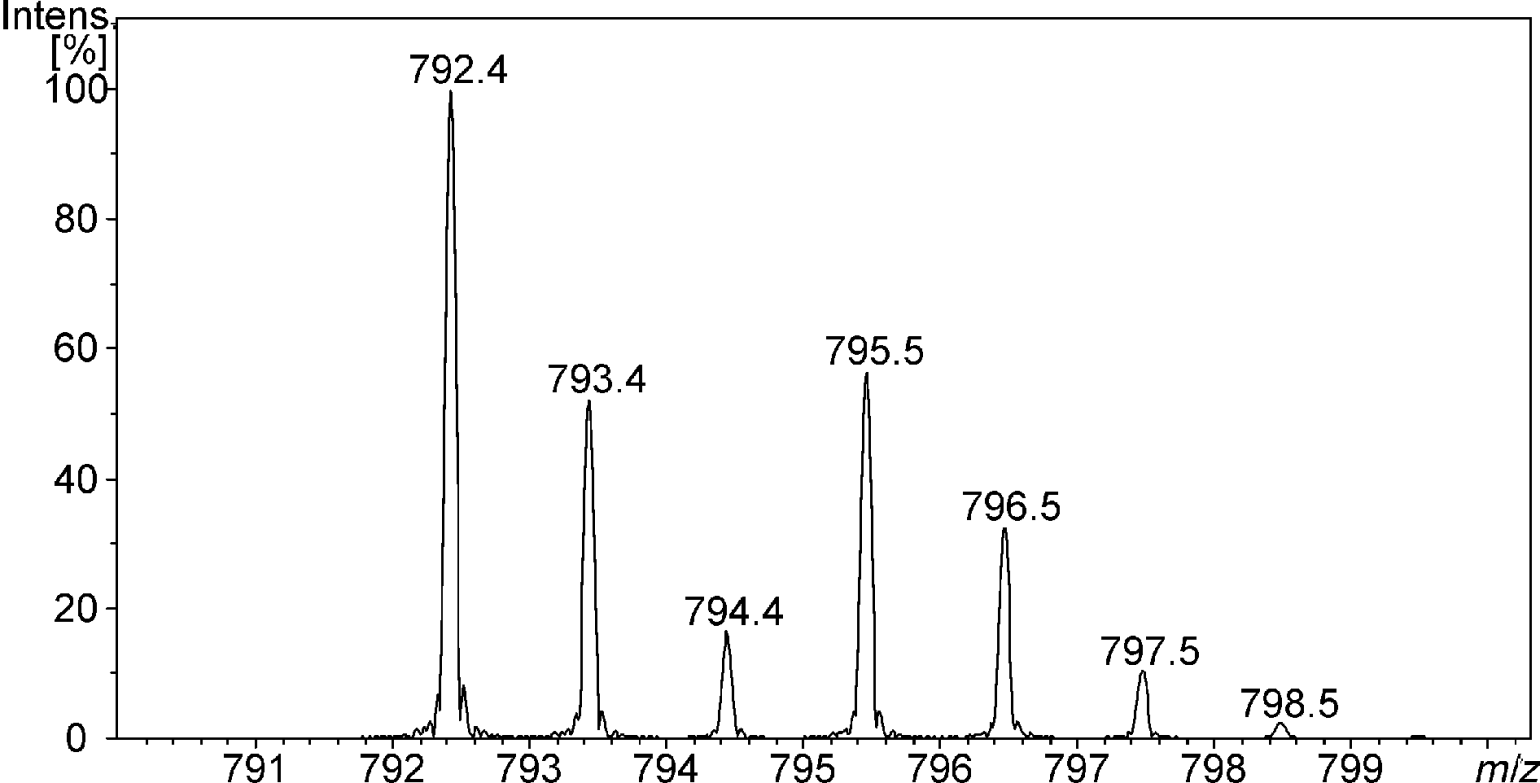
ESI-mass spectrum (positive mode) of a 1:1:1 mixture of **1**, protonated L-leucine methyl ester (LeuOMe), and protonated L-isoleucine trideuteromethyl ester (IleOMe-*d*_3_) in a 1:1 mixture of CH_2_Cl_2_/MeOH (*m*/*z* = 792.4 LeuOMe–**1**, *m*/*z* = 795.5 IleOMe-*d*_3_–**1**).

In our case, we used the methyl groups of the ester function as the mass label by employing either the normal protiated methyl group or a trideuteromethyl group as the labelled one. To test the relative affinity of a receptor towards two isomeric substrates we prepared solutions that contain 1:1:1 mixtures of the receptor, a non-labelled guest, and an isotopically-labelled guest. These solutions were analysed by ESI-mass spectrometry (Figure 4). The intensity ratios of the signals of the host–guest complexes can then be used to conclude which guest is bound stronger since the mass difference is large enough to allow an individual detection but also small enough not to cause any problems due to mass discrimination phenomena.

It is important to note that these measurements are obviously not biased by, e.g., different solvation energies of the structural isomers which could cause different ESI response factors because we did not observe different intensity ratios when we changed the overall concentration of our samples. Another important point that has to be mentioned here, however, is that intensity differences of the complexes might also be the result of differences in the tendency to dissociate under the conditions of the ESI–MS experiment. Unfortunately, the low mass of the leucine derivatives investigated here did not allow to study this phenomenon directly because the FTICR spectrometer we used is tuned in a way that it does not allow detection of such low molecular mass ions with the necessary accuracy. Hence, we optimized our ESI conditions using larger and well-detectable protonated amino acid esters like protonated phenylalanine benzyl ester to make sure that the conditions are mild enough not to cause dissociation.

**Figure 5:**
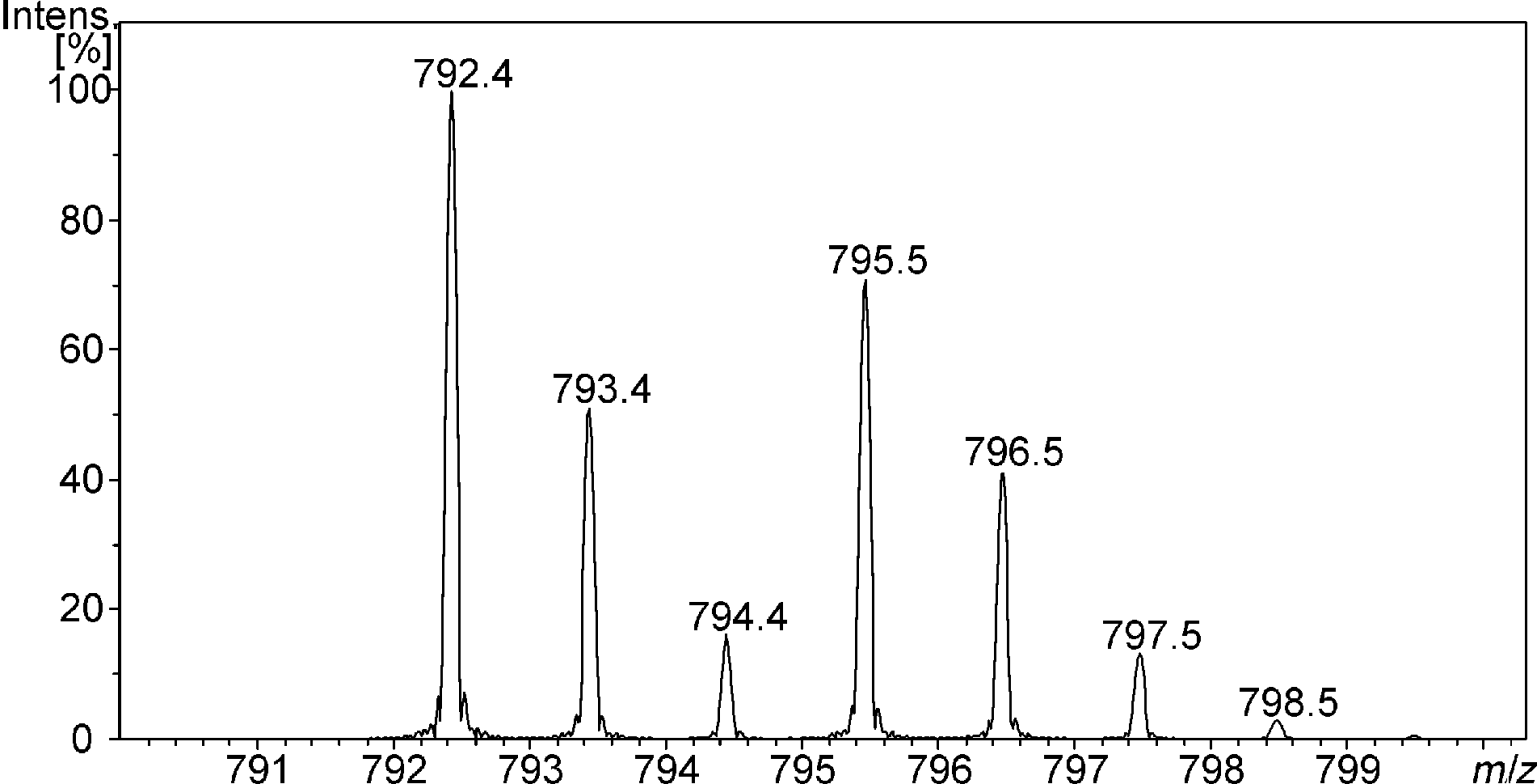
ESI-mass spectrum (positive mode) of a 1:1:1 mixture of **1**, protonated L-leucine methyl ester (LeuOMe), and protonated L-leucine trideuteromethyl ester (LeuOMe-*d*_3_) in a 1:1 mixture of CH_2_Cl_2_/MeOH (*m*/*z* = 792.4 LeuOMe–**1**, *m*/*z* = 795.5 LeuOMe-*d*_3_–**1**).

Having made sure that the method is in principle suitable to study the recognition of protonated amino acid derivatives in a competitive fashion there is still one more factor that has to be taken into account: non-covalent isotope effects [23] might also cause significant differences of the signals’ intensities. Therefore, every experiment has to be repeated with the different order of isotope labelling and in addition one should measure the same isomer in both forms – non-labelled and labelled – in order to quantify this effect (Figure 5).

Table 1 lists the results of the ILGM measurements with regard to the affinity of templates **1** and **2** towards L-norleucine, L-isoleucine, and L-leucine that were calculated according to Equation 1.

[1]
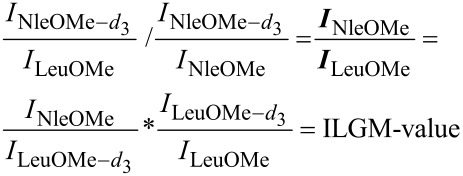


**Table 1:** ILGM ratios for the recognition of the three pairs of L-leucine isomers by templates **1** and **2**.

pair of isomers	**1**	**2**

L*-*NleOMe/L-IleOMe	1.5	1.2
L*-*IleOMe/L-LeuOMe	0.7	0.8
L*-*NleOMe/L-LeuOMe	1.0	0.9

These ratios translate into the following relative affinities of our receptors:

Receptor **1**: L-NleOMe ≈ L-LeuOMe > L-IleOMe

Receptor **2**: L-LeuOMe ≥ L-NleOMe > L-IleOMe

**Figure 6:**
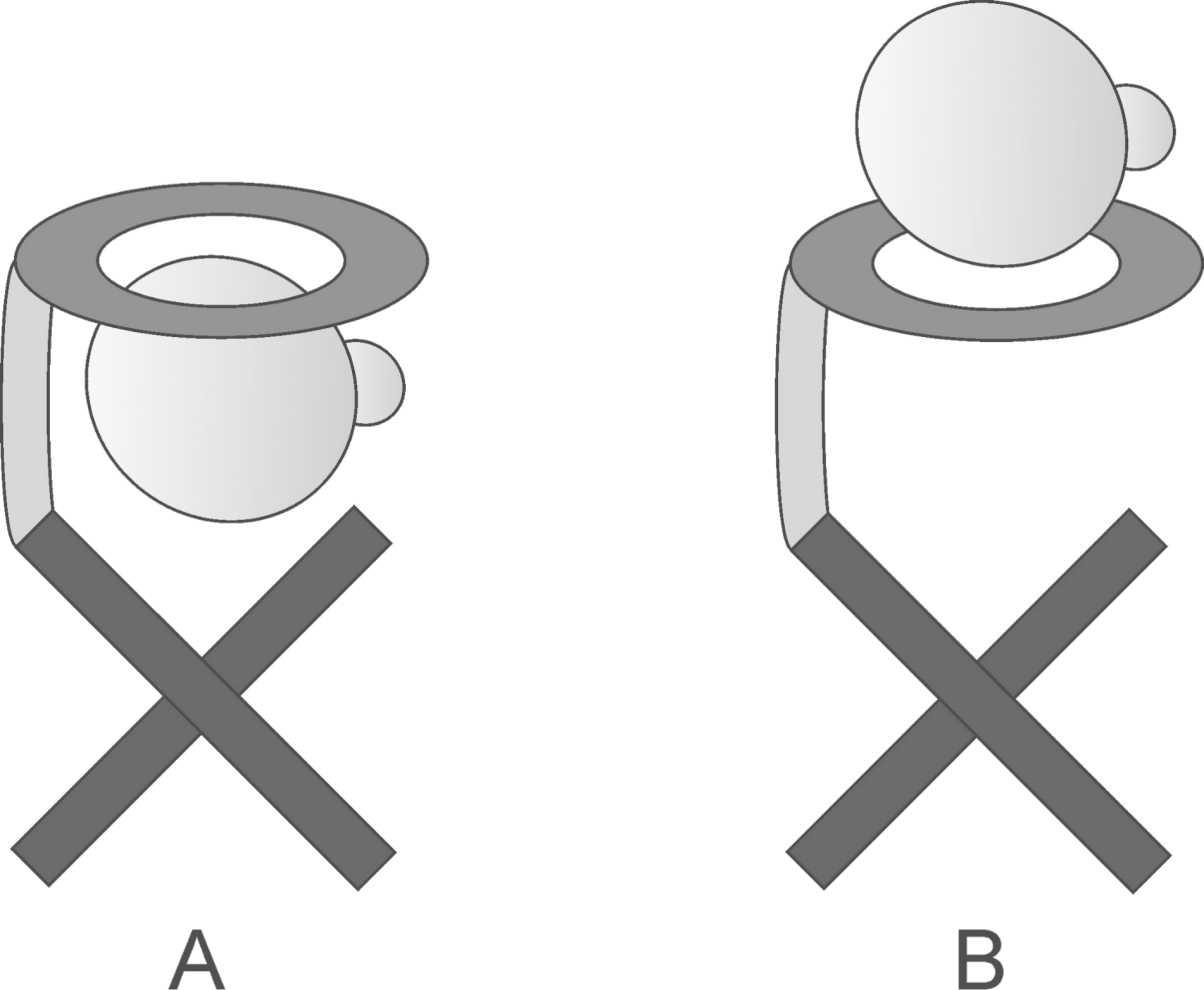
Two different motifs for the binding of substrates to the templates.

Interestingly, isoleucine methyl ester is the worst guest in both cases although the effect is not large enough yet to think about an application for the separation of the isomers on a larger scale via supramolecular transport, for instance. However, these results are still promising with regard to the development of a template that allows the efficient separation of isoleucine and leucine which is obviously the most interesting challenge. The non-covalent isotope effects clearly demonstrate that the ester group of the amino acid derivative must interact with our templates, and therefore, these effects tell us something about the relative spatial orientation of the template and the substrate within the host–guest complexes (Figure 6).

In fact, the deuterated guests were always bound less good than the protiated guests. This can be rationalized by assuming attractive dispersive interaction to play a significant role during the recognition event. The deuterated group is on average slightly smaller than the protiated group [14–26] which causes a smaller van der Waals volume of the deuterated group [27–32]. In a first approximation, however, the dispersive interactions get stronger with an increasing size of the volume and consequently the contact area, and hence, the deuterated compounds ability to interact via those interactions is weaker [33–35]. This observation perfectly agrees with the conclusion that the ester group gets into close contact with the non-polar parts of the templates, and thereby, contribute to the binding affinity by attractive dispersive interactions.

Hence, we can conclude that the binding of the amino acid derivatives occurs in a way that puts the substrates in close contact to the crown ether and the spirobifluorene backbone as depicted in motif A in Figure 6 rather than placing them in a more remote position where only the ammonium group can interact with the crown ether moiety which would largely rule out any isotope effect (motif B in Figure 6).

## Conclusion

We have synthesised two new functionalized crown ethers **1** and **2** both bearing a 9,9’-spirobifluorene moiety and studied their ability to differentiate between the constitutional isomers L-leucine, L-norleucine, and L-isoleucine. ESI-mass spectrometry measurements using the isomer labelled guest method (ILGM) were used for this purpose. This technique proved to be a quick method that needs only trace amounts of material and allows easily studying relative affinities in competitive experiments. Interestingly, L-isoleucine was found to be the worst guest for both of our templates, whereas L-leucine and L-norleucine turned out to be almost equally good guests. The occurrence of significant non-covalent isotope effects allowed us to obtain information on the relative spatial orientation of the substrates within the concave host structure. Obviously, the spirobifluorene part of **1** and **2** provides a possibility to undergo attractive dispersive interactions with non-polar parts of the substrates. Nevertheless, however, it also provides enough rigidity to differentiate between different substitutions in the β-position of the amino acid derivative at the same time. Hence, we think it is not fallacious that **1** and **2** might serve as kind of lead structures to develop even better templates in the future.

## Supporting Information

Experimental data of all new compounds and of the ESI mass spectrometric experiments.

File 1 Experimental data, ESI-mass spectrometric experiments.
