# Supplementary material for: Molecular recognition of isomeric protonated amino acid esters monitored by ESI-mass spectrometry
Source: Beilstein J Org Chem. 2014 Apr 9;10:825–31. doi: 10.3762/bjoc.10.78 (PMC3999762; doi:10.3762/bjoc.10.78)
Supplement: File 1 — Experimental data, ESI-mass spectrometric experiments. [file Beilstein_J_Org_Chem-10-825-s001.pdf]

Supporting Information

for

Molecular recognition of isomeric protonated amino acid esters monitored by ESI-mass spectrometry

Andrea Liesenfeld and Arne Lützen*

Address: University of Bonn, Kekulé-Institute of Organic Chemistry and Biochemistry, Gerhard-Domagk-Str.1, D-53121 Bonn, Germany

Email: Arne Lützen - arne.luetzen@uni-bonn.de

* Corresponding author

Experimental part and ESI-mass spectrometric experiments

Experimental

General remarks: All solvents were distilled and dried prior to use according to standard procedures. All syntheses with air- and moisture-sensitive compounds were performed under Schlenk conditions with argon. Column chromatography was performed on Macherey-Nagel silica gel 60 M (0.04–0.063 mm) or Merck aluminium oxide. All solvents used as eluents for column chromatography were distilled prior to use. ^{1}H and ^{13}C NMR spectra were recorded at 298 K on Bruker Avance 300 (^{1}H: 300.1 MHz, ^{13}C: 75.5 MHz), AM 400 (^{1}H: 400.1 MHz, ^{13}C: 100.6 MHz) or AM 500 (^{1}H: 500.1 MHz, ^{13}C: 125.8 MHz) spectrometers. ^{1}H NMR chemical shifts are reported as δ values (ppm) relative to residual non-deuterated solvent as the internal standard. ^{13}C NMR chemical shifts are given in δ values (ppm) relative to the deuterated solvent as the internal standard. Mass spectra were taken on a Thermo Finnigan MAT 95 XL, Mat 90 (EI, Hi-Res-EI) or a Bruker micrOTOF-Q (ESI, Hi-Res-ESI). The ILGM measurements were performed on a Bruker Apex IVFT-ICR. Chemicals and reagents (except for the solvents) obtained from commercial sources were used as received. The following compounds were prepared according to published procedures: 2-bromo-4'-methoxybiphenyl (**3**) [1,2], (1,4,7,10,13,16-hexaoxacyclooctadecan-2-yl)methyl 4-methylbenzenesulfonate (**10**) [3,4].

9-(4’-Methoxybiphenyl-2-yl)-9*H*-fluoren-9-ol (4): Under an argon atmosphere magnesium (699 mg, 28.8 mmol) and a spatula tip of iodine in anhydrous THF (10 mL) were heated for one hour to reflux. After cooling to room temperature a

1 Li, C.-W.; Wang, C.-I.; Liao, H.-Y.; Chaudhuri, R.; Liu, R.-S. *J. Org. Chem.* **2007**, *72*, 9203-9207.
2 Varela, J. A.; Peña, D.; Goldfuss, B.; Denisenko, D.; Kulhanek, J.; Polborn, K.; Knochel, P. *Chem. Eur. J.* **2004**, *10*, 4252-4264.
3 Czech, B.; Son, B.; Bartsch, R. A. *Tetrahedron Lett.* **1983**, *24*, 2923-2926.
4 Dishong, D. M.; Diamond, C. J.; Cinoman, M. I.; Gokel, G. W. *J. Am. Chem. Soc.* **1983**, *105*, 586-593.

solution of 2-bromo-4'-methoxybiphenyl (**3**, 4.893 g, 18.6 mmol) in anhydrous THF (20 mL) was added. The resulting mixture was heated to 55 °C for two hours. A solution of 9-fluorenone (9.509 g, 52.8 mmol) in anhydrous THF (30 mL) was slowly added and stirred for 20 hours at 55 °C. The reaction mixture was poured into an ice-cold sodium solution of 10% HCl and the resulting mixture was stirred for 1 hour at room temperature. The mixture was extracted several times with CH_2Cl_2. The combined organic layers were washed subsequently with sat. $NaHCO_3$ solution, sat. $Na_2S_2O_3$ solution, and with brine and then dried with Na_2SO_4. The solvent was removed under reduced pressure. The crude product was recrystallized from ethanol, and the product was obtained as a white solid (3.718 g, 10,2 mmol).

^{1}H NMR (500.1 MHz, $CDCl_3$, 293 K) δ[ppm] = 3.65 (s, 3 H); 5.89 (ddd, 2 H, J= 9.5 Hz, J = 8.8 Hz, J = 2.8 Hz); 6.13 (ddd, 2 H, J = 9.5 Hz, J = 8.8 Hz, J = 2.2 Hz); 6.89 (dd, 1 H, J = 8.0 Hz, J = 1.4 Hz); 7.11 – 7.21 (m, 8 H); 7.29 (ddd, 1 H, J= 8.0 Hz, J = 7.6 Hz, J = 1.2 Hz); 7.50 (ddd, 1 H, J = 7.6 Hz, J = 7.9 Hz, J = 1.4 Hz), 8.43 (dd, 1 H, J = 7.9 Hz, J = 1.2 Hz). ^{13}C NMR (100.6 MHz, $CDCl_3$, 293 K) δ[ppm] = 55.3, 82.6, 111.9, 120.1, 124.4, 126.4, 127.0, 127.2, 128.0, 128.8, 130.0, 131.7, 133.1, 140.3, 150.8, 157.2. EI HRMS: *m/z* calculated for $C_{26}H_{20}O_2$ 364.1463, found: 364.1464.

2-Methoxy-9,9'-spirobifluorene (5): A solution of 9-(4'-methoxybiphenyl-2-yl)-9*H*-fluoren-9-ol (3.77 g, 10.4 mmol) in conc. acetic acid (15 mL) was heated to reflux. Conc. HCl was added. An off-white precipitate formed directly after addition of the first portion of hydrochloric acid. More HCl was added until no precipitation occurred anymore (total amount of added HCl approx. 40 mL). The resulting mixture was heated for 20 minutes to reflux. After cooling to room temperature H_2O (18 mL) was added. The precipitate was filtered off and washed with H_2O. The yellow/brown crude product was dried under high vacuum and then dissolved in CH_2Cl_2. To remove the

remaining acetic acid the solution was washed with saturated Na_2CO_3 solution, H_2O, and brine and then dried with Na_2SO_4. The solvent was removed under reduced pressure and the crude product was subjected to column chromatography on silica gel using a 1:1 mixture of toluene/cyclohexane as eluent to give the desired product as a white solid (3.43 g, 9.9 mmol).

^1H NMR (300.1 MHz, $CDCl_3$, 293 K) δ[ppm] = 3.63 (s, 3 H); 6.26 (d, 1 H, J= 2.3 Hz); 6.67 (dd, 1 H, J= 7.5 Hz, J= 1.0 Hz); 6.77 (dd, 2 H, J = 7.5 Hz, J = 1.0 Hz); 6.91 (dd, 1 H, J = 8.4 Hz, J= 2.3 Hz); 7.01 (ddd, 1 H, J = 7.5 Hz, J = 7.4 Hz, J = 1.0 Hz); 7.10 (ddd, 2 H, J = 7.5 Hz, J = 7.4 Hz, J = 1.0 Hz); 7.30 – 7.41 (m, 3 H); 7.74 (dd, 2 H, J = 8.4 Hz, J = 0.4 Hz); 7.84 (dd, 2 H, J = 7.5 Hz, J = 0.9 Hz). ^{13}C NMR (75.5 MHz, $CDCl_3$, 293 K) δ[ppm] = 55.4, 65.9, 109.3,113.9, 119.1, 119.9, 120.7, 123.8, 124.1, 126.6, 127.6, 127.7, 127.8, 134.7, 141.7, 148.4, 148.9, 150.5, 159.9. EI HRMS: *m/z* calculated for $C_{26}H_{18}O$ 346.1361, found: 346.1358.

2-Hydroxy-9,9'-spirobifluorene (6): Under an argon atmosphere 2-methoxy-9,9'-spirobifluorene (**5**) (1.23 g, 3.56 mmol) was dissolved in anhydrous CH_2Cl_2 (10 mL) and cooled down to −78 °C. A 1 M solution of boron tribromide (12 mL, 12 mmol) in CH_2Cl_2 was added dropwise. After completion the reaction mixture was warmed to room temperature and stirred for 16 hours. After adding H_2O (40 mL) for hydrolysis and ethyl acetate (40 mL), the reaction mixture was neutralized with 6 M NaOH solution. The crude product was extracted several times with ethyl acetate, dried over Na_2SO_4 and concentrated in vacuum to give the crude product, which was purified by column chromatography (silica gel, eluent: toluene). A white solid was obtained (1.17 g, 3.52 mmol).

^{1}H NMR (300.1 MHz, $CDCl_3$, 293 K) δ[ppm] = 4.86 (s, 1 H); 6.17 (d, 1 H, J = 2.4 Hz); 6.70 (dd, 1 H, J = 7.6 Hz, J = 1.0 Hz); 6.76 (dd, 2 H, J = 7.6 Hz, J = 0.9 Hz); 6.84 (dd, 1H, J = 8.3 Hz, J = 2.4 Hz); 7.04 (ddd, 1 H, J = 7.5 Hz, J = 7.6 Hz, J = 1.0 Hz); 7.12 (ddd, 2 H, J = 7.5 Hz, J = 7.4 Hz, J=0.9 Hz); 7.34 (ddd, 1 H, J = 7.6 Hz, J = 7.5 Hz, J = 1.0 Hz); 7.37 (ddd, 2 H, J = 7.6 Hz, J = 7.4 Hz, J = 0.9 Hz); 7.70 (d, 1 H, J = 8.3 Hz); 7.74 (dd, 1 H, J = 7.6 Hz, J = 1.0 Hz); 7.84 (dd, 2 H, J= 7.5 Hz, J = 0.9 Hz). ^{13}C NMR (75.5 MHz, $CDCl_3$, 293 K) δ[ppm] = 65.8, 111.0, 115.0, 119.1, 119.9, 121.0, 123.9, 124.1, 126.7, 127.6, 127.7, 127.8, 134.7, 141.6, 148.1, 148.8, 150.8, 155.7. EI HRMS: *m/z* calculated for $C_{25}H_{16}O$: 332.1201, found: 332.1201.

(9,9'-Spirobifluorene-2-yl)trifluoromethanesulfonate (7): Under an argon atmosphere a solution of 2-hydroxy-9,9'-spirobifluorene (4.04 g, 12.1 mmol) in anhydrous triethylamine (24 mL, 0.171 mol) and anhydrous CH_2Cl_2 (80 mL) was cooled down to −30 °C and a solution of triflic anhydride (12.5 mL, 72.5 mmol) in anhydrous CH_2Cl_2 (20 mL) was slowly added dropwise. After complete addition the reaction mixture was warmed to rt and stirred for 16 hours. Then the reaction mixture was poured into 150 mL H_2O and extracted several times with CH_2Cl_2. The combined organic layers were washed with brine, dried with Na_2SO_4, and concentrated in vacuum. The crude product was purified by column chromatography on silica gel (eluent: toluene/cyclohexane 1:1). The product was obtained as a yellow, amorphous solid (3.62 g, 7.80 mmol).

^{1}H NMR (400.1 MHz, $CDCl_3$, 293 K) δ[ppm] = 6.60 (d, 1 H, J = 2.4 Hz); 6.72 (dd, 2 H, J = 7.6 Hz, J = 1.5 Hz); 6.75 (dd, 1 H, J = 7.7 Hz, J = 1.5 Hz); 7.11 – 7.21 (m, 3H); 7.30 (dd, 1 H, J = 8.4 Hz, J = 2.4 Hz); 7.37 – 7.43 (m, 3 H); 7.82 – 7.90 (m, 4 H). ^{13}C NMR (100.6 MHz, $CDCl_3$, 293 K) δ[ppm] = 21.5, 66.0, 117.4, 120.2, 120.3, 121.0,

121.1, 123.9, 124.2, 128.1, 128.2, 128.7, 129.0, 139.6, 141.8, 142.0, 147.3, 148.9, 149.2, 151.3. EI HRMS: *m/z* calculated for $C_{26}H_{15}F_3O_3S$: 464.0694, found: 464.0694.

3-(9,9'-Spirobifluorene-2-yl)prop-2-yn-1-ol (8): Under an argon atmosphere 2-(9,9'-spirobifluorene-2-yl)trifluoromethanesulfonate (**7**, 200 mg, 0.43 mmol), $Pd(PPh_3)_2Cl_2$ (17 mg, 5 mol %), and CuI (5 mg, 5 mol %) were dissolved in anhydrous DMF (13 mL) and stirred for 5 min at rt. Then a solution of prop-2-ynol (30 µL) in anhydrous triethylamine (5 mL) was added. The reaction mixture was stirred at 40 °C for 16 hours. Then the reaction was quenched with brine and repeatedly extracted with CH_2Cl_2. The combined organic layers were washed with $NaHCO_3$ solution, H_2O, and brine and dried with Na_2SO_4. The solvent was removed under reduced pressure. The crude product was purified by column chromatography on silica gel using cyclohexane/ethyl acetate 3:1 as eluent to give the desired product as a yellow solid (151 mg, 0.41 mmol).

^{1}H NMR (500.1 MHz, $CDCl_3$, 293 K) δ[ppm] = 4.36 (s, 2 H); 6.73 (dd, 2 H, J = 7.6 Hz, J = 0.8 Hz); 6.76 (dd, 1 H, J = 7.6 Hz, J = 0.7 Hz); 6.82 (d, 1 H, J = 1.2 Hz); 7.10 – 7.15 (m, 3 H); 7.35 – 7.40 (m, 3 H); 7.45 (dd, 1 H, J = 7.9 Hz, J = 1.2 Hz); 7.79 (dd, 2 H, J = 7.9 Hz, J = 0.4 Hz); 7.83 – 7.86 (m, 3 H). ^{13}C NMR (125.8 MHz, $CDCl_3$, 293 K) δ[ppm] = 51.5, 65.7, 86.0, 87.4, 119.9, 120.0, 120.3, 124.0, 124.1, 127.4, 127.9 – 128.9, 131.3, 141.0, 141.7, 142.1, 148.1, 148.9, 149.0. EI HRMS: *m/z* calculated for $C_{28}H_{18}O$: 370.1358, found: 370.1357.

2-[4-(Methoxy)phenyl]-9,9'-spirobifluorene: Under an argon atmosphere 2-(9,9'-spirobifluorene-2-yl)trifluoromethanesulfonate (**7**) (984 mg, 2.12 mol), 4-methoxy-phenylboronic acid (382 mg, 2.51 mol), cesium fluoride (1.64 g, 10.8 mol), and $Pd(PPh_3)_4$ (143 mg, 0,12 mol) were dissolved in anhydrous THF (50 mL). The

reaction mixture was heated under reflux for 16 hours. After cooling to rt the solution was suspended in CH_2Cl_2 and washed with H_2O, dried with Na_2SO_4 and concentrated in vacuum to give the crude product. This was purified by column chromatography on silica gel (eluent: CH_2Cl_2/cyclohexane 2:3) to give the desired product as a white solid (780 mg, 1.91 mol).

^1H NMR (400.1 MHz, $CDCl_3$, 293 K) δ[ppm] = 3.78 (s, 3 H), 6.73 (dd, 1 H, J = 7.5 Hz, J = 1.6 Hz); 6.79 (ddd, 2 H, J = 7.6 Hz, J = 0.8 Hz, J = 0.7 Hz); 6.85 (ddd, 2 H, J = 8.8 Hz, J = 1.6 Hz, J = 2.1 Hz); 6.91 (dd, 1 H, J = 1.7 Hz, J = 0.3 Hz); 7.08 – 7.14 (m, 3 H); 7.34 – 7.40 (m, 5 H); 7.58 (dd, 1 H, J = 7.9 Hz, J = 1.7 Hz); 7.84 – 7.90 (m, 4 H). ^{13}C NMR (100.6 MHz, $CDCl_3$, 293 K) δ[ppm] = 26.9, 66.0, 114.0, 119.9, 120.0, 120.2, 122.2, 124.0, 124.1, 126.4, 127.6 – 127.8, 128.0, 133.5, 140.4, 140.5, 141.5, 141.8, 148.8, 149.0, 149.4, 159.0. EI HRMS: *m/z* calculated for $C_{32}H_{22}O_2$: 422.1671, found: 422.1670.

4-(9,9'-Spirobifluorene-2-yl)phenol (9): Under an argon atmosphere 2-(4-methoxyphenyl)-9,9'-spirobifluorene (458 mg, 1.08 mmol) was dissolved in anhydrous CH_2Cl_2 (60 mL) and cooled to −78 °C. A 1 M solution of boron tribromide in CH_2Cl_2 (3.3 mL, 3.3 mmol) was added dropwise. The reaction mixture was warmed to rt and stirred for 16 hours. Thereafter, H_2O (100 mL) was added to hydrolyse excess boron tribromide and ethyl acetate (100 mL) was added. Then the mixture was neutralized with 6 M NaOH solution, extracted several times with ethyl acetate, dried with Na_2SO_4, and concentrated in vacuum. The crude product was purified by column chromatography on silica gel with a 1:1 mixture of cyclohexane and ethyl acetate as eluent, to give the desired product as a white solid (441 mg, 1.08 mmol).

^{1}H NMR (400.1 MHz, $CDCl_3$, 293 K) δ[ppm] = 6.73 (dd, 1 H, J = 7.7 Hz, J = 0.8 Hz); 6.75 – 6.81 (m, 4 H); 6.89 (dd, 1 H, J = 1.7 Hz, J = 0.4 Hz); 7.07 – 7.14 (m, 3 H); 7.30 (ddd, 2 H, J = 8.8 Hz, J = 3.0 Hz, J = 2.2 Hz); 7.37 (ddd, 3 H, J = 7.5 Hz, J = 1.0 Hz, J = 0.9 Hz); 7.56 (dd, 1 H, J = 8.0 Hz, J = 1.7 Hz); 7.83 – 7.89 (m, 4 H). ^{13}C NMR (100.6 MHz, $CDCl_3$, 293 K) δ[ppm] = 66.0, 115.4, 119.9, 120.0, 120.2, 122.2, 124.0, 124.1, 126.3, 127.6 – 127.8, 128.3, 133.6, 140.5, 141.4, 141.8, 148.8, 149.0, 149.4, 155.0. EI HRMS: *m/z* calculated for $C_{31}H_{19}O$: 407.1436, found: 407.1438.

2-((3-(9,9'-Spirobifluorene-2-yl)prop-2-ynyloxy)methyl)-1,4,7,10,13,16-hexaoxa-cyclooctadecane (crown ether 1): Under an argon atmosphere 3-(9,9'-spirobifluorene-2-yl)prop-2-yn-1-ol (**8**, 122 mg, 0,33 mmol) was dissolved in anhydrous THF (15 mL) and cooled down to 0 °C. Then NaH (60% in mineral oil) (79 mg, 2.0 mmol) was added. The reaction mixture was warmed to rt and stirred for one hour. A solution of (1,4,7,10,13,16-hexaoxacyclooctadecan-2-yl)methyl 4-methyl-benzenesulfonate (**10**) in THF (5 mL) was added and stirred for 16 hours at rt. The mixture was quenched with methanol and the solvents were removed under reduced pressure. The residue was dissolved in CH_2Cl_2 and washed with distilled water. NaCl was added to improve phase separation. The organic layer was washed again with brine and dried with Na_2SO_4. The solvent was removed again under reduced pressure. The crude product was purified by column chromatography on neutral aluminium oxide using a gradient of a 1:1 mixture of cyclohexane/ethyl acetate → pure ethyl acetate as eluent. The product was obtained as a white solid (53 mg, 8.2 mmol).

^{1}H NMR (400.1 MHz, $CDCl_3$, 293 K) δ[ppm] = 3.55 – 3.79 (m, 23 H); 4.28 (s, 2 H); 6.70 – 6.75 (m, 3 H); 6.81 (d, 1 H, J = 1.3 Hz); 7.08 – 7.14 (m, 3 H); 7.34 – 7.39 (m, 3

H); 7.45 (dd, 1 H, J = 7.7 Hz, J = 1.3 Hz); 7.78 (dd, 1 H, J = 7.8 Hz, J = 0.5 Hz); 7.81 – 7.85 (m, 3 H). ^{13}C NMR (100.6 MHz, $CDCl_3$, 293 K) δ[ppm] = 59.3, 65.8, 69.9 – 71.6, 78.3, 85.5, 86.6, 120.0, 120.2, 120.4, 124.1, 124.2, 127.5, 127.9 – 128.1, 131.6, 141.1, 141.8, 142.1, 148.2, 149.0, 149.2. EI HRMS: *m/z* calculated for $C_{41}H_{42}O_7Na^+$: 669.2823, found: 669.2818.

2-((4-(9,9'-Spirobifluorene-2-yl)phenoxy)methyl)-1,4,7,10,13,16-hexaoxacyclo-octadecane (crown ether 2): Under an argon atmosphere 4-(9,9'-spirobifluorene-2-yl)phenol (**9**, 210 mg, 0.51 mmol) was dissolved in anhydrous THF (15 mL) and cooled to 0 °C. Then NaH (60% in mineral oil, 68 mg, 1.7 mmol) was added. The ice bath was removed and the reaction mixture was stirred for one hour at rt. After that time (1,4,7,10,13,16-hexaoxacyclooctadecan-2-yl)methyl 4-methylbenzenesulfonate (**10**, 129 mg, 0.29 mmol) dissolved in THF (8 mL) was added and stirred overnight at rt. The mixture was quenched with methanol and the solvents were removed under reduced pressure. The residue was dissolved in CH_2Cl_2 and washed with distilled water. NaCl was added to improve phase separation. The organic layer was washed again with brine and dried with Na_2SO_4. The solvent was removed again under reduced pressure. The crude product was purified by column chromatography on neutral aluminium oxide using a gradient of a 1:1 mixture of cyclohexane/ethyl acetate → pure ethyl acetate as eluent. The product was obtained as a white solid (151 mg, 0.22 mmol).

^1H NMR (400.1 MHz, $CDCl_3$, 293 K) δ[ppm] = 3.60 – 4.06 (m, 25 H); 6.72 (d, 1 H, J = 7.5 Hz); 6.78 (d, 2 H, J = 7.6 Hz); 6.85 (dd, 2 H, J = 8.4 Hz, J = 1.6 Hz); 6.90 (d, 1 H, J = 1.2 Hz); 7.07 – 7.14 (m, 3 H); 7.31 – 7.40 (m, 5 H); 7.58 (dd, 1 H, J = 8.0 Hz, J = 1.7 Hz); 7.83 – 7.89 (m, 4 H). ^{13}C NMR (100.6 MHz, $CDCl_3$, 293 K) δ[ppm] = 66.1,

68.2, 70.1 – 71.3, 77.8, 114.9, 120.0, 120.1, 120.3, 122.3, 124.1, 124.3, 126.5, 127.7 – 128.0, 128.1, 133.7, 140.6, 141.6, 141.9, 148.9, 149.2, 149.5, 158.4. EI HRMS: *m/z* calculated for $C_{44}H_{44}O_7Na^+$: 707.2979, found: 707.2975.

General procedure for the synthesis of amino acid deuteromethyl ester hydrochlorides: Under an argon atmosphere 5 mmol of an amino acid were dissolved in 10 mL of deuterated methanol and cooled to 0 °C. Then 0.6 mL of freshly distilled thionyl chloride were added dropwise. After complete addition the reaction solution was heated to reflux for 24 hours. Then it was allowed to cool down to rt, the solvent was removed under reduced pressure to obtain the product which was used without any further purification.

L-Leucine-trideuteromethyl ester hydrochloride: 903 mg, 4.9 mmol. ^{1}H NMR (300.1 MHz, $CDCl_3$, 293 K) δ[ppm] = 0.99 (q, 6 H); 1.64–1.89 (m, 3 H); 4.03 (t, 1 H) ^{13}C NMR (75.5 MHz, $CDCl_3$, 293 K) δ[ppm] = 22.3, 22.5, 25.6, 40.7, 52.5, 171.4; melting point 135-140 °C, $[\alpha]_D^{20}$ = +11.1° (c = 3.03, H_2O).

L-Isoleucine-trideuteromethyl ester hydrochloride: 873 mg, 4.7 mmol. ^{1}H NMR (400.1 MHz, $CDCl_3$, 293 K) δ[ppm] = 0.96–1.03 (m, 6 H); 1.30–1.60 (m, 2 H); 1.95–2.04 (m, 1 H); 3.83 (s, 3 H); 4.01 (d, 1H, J= 3.9 Hz). ^{13}C NMR (100.6 MHz, $CDCl_3$, 293 K) δ[ppm] = 11.6, 14.5, 26.3, 37.4, 53.0, 57.9, 169.9; melting point 74-78 °C, $[\alpha]_D^{20}$ = +21.0° (c = 3.19, H_2O).

L-norleucine-trideuteromethyl ester hydrochloride: 848 mg, 4.6 mmol. ^{1}H NMR (400.1 MHz, $CDCl_3$, 293 K) δ[ppm] = 0.92–0.97 (m, 3 H); 1.32–1.47 (m, 4 H); 1.83–

1.97 (m, 2 H); 4.01–4.06 (m, 1 H). ^{13}C NMR (100.6 MHz, $CDCl_3$, 293 K) δ[ppm] = 13.6, 22.8, 27.6, 30.8, 53.6, 170.7; melting point 137-141 °C, $[\alpha]_D^{20}$ = +16.5° (c = 2.79, H_2O).

General procedure for the ILGM ESI-MS measurements: Stock solutions (1 mM) of **1**, **2**, and all amino acid guests in a 1:1 mixture of CH_2Cl_2/MeOH were prepared. Aliquots of 50 µL of these solutions were mixed to obtain 1:1:1 mixtures of template **1** or **2**, the non-labelled, and the labelled substrate which were diluted with the same solvent mixture to a total sample volume of 1 mL. Mass spectra of these solutions were recorded on a Bruker Apex IV Fourier-transform ion-cyclotron resonance (FT-ICR) mass spectrometer with a 7.05 T magnet and an Apollo ESI ion source with a 70° off-axis spray needle. The solutions were introduced into the ion source with a syringe pump at flow rates of 200 µL/h. The ion transfer into the first of three differential pump stages in the ion source occurred through a glass capillary with nickel coating at both ends and a 0.5 mm inner diameter. Ionisation parameters were adjusted as follows: capillary voltage: −3380 V, end plate voltage: −3300 V, cap exit voltage: 150 V, skimmer 1 voltage: 15 V, skimmer 2 voltage: 10 V, and temperature of drying gas: 200 °C. Spectra are a summation of 128 scans. Every sample was measured twice to calculate averaged values.

Furthermore a series of experiments was performed with samples of lower and higher concentrations (aliquots of 10 µL or 250 µL of the stock solutions) to rule out effects based on different ESI response factors caused by e.g. different desolvation energies.
